# Reliability of Sonoelastography Measurement of Tongue Muscles and Its Application on Obstructive Sleep Apnea

**DOI:** 10.3389/fphys.2021.654667

**Published:** 2021-03-25

**Authors:** Cheng-An Chu, Yunn-Jy Chen, Ke-Vin Chang, Wei-Ting Wu, Levent Özçakar

**Affiliations:** ^1^Department of Dentistry, School of Dentistry, National Taiwan University Hospital, Taipei, Taiwan; ^2^Department of Physical Medicine and Rehabilitation and Community and Geriatric Research Center, National Taiwan University Hospital, Bei-Hu Branch and National Taiwan University College of Medicine, Taipei, Taiwan; ^3^Center for Regional Anesthesia and Pain Medicine, Wang-Fang Hospital, Taipei Medical University, Taipei, Taiwan; ^4^Department of Physical and Rehabilitation Medicine, Hacettepe University Medical School, Ankara, Turkey

**Keywords:** sleep apnea, ultrasound, elastography, shear wave, tongue

## Abstract

Few studies have explored the feasibility of shear-wave ultrasound elastography (SWUE) for evaluating the upper airways of patients with obstructive sleep apnea (OSA). This study aimed to establish a reliable SWUE protocol for evaluating tongue muscle elasticity and its feasibility and utility in differentiating patients with OSA. Inter-rater and intra-rater reliability of SWUE measurements were tested using the intraclass correlation coefficients. Submental ultrasound was used to measure tongue thickness and stiffness. Association between the ultrasound measurements and presence of OSA was analyzed using multivariate logistic regression. One-way analysis of variance was used to examine if the values of the ultrasound parameters varied among patients with different severities of OSA. Overall, 37 healthy subjects and 32 patients with OSA were recruited. The intraclass correlation coefficients of intra‐ and inter-rater reliability for SWUE for tongue stiffness ranged from 0.84 to 0.90. After adjusting for age, sex, neck circumference, and body mass index, the risk for OSA was positively associated with tongue thickness [odds ratio 1.16 (95% confidence interval 1.01–1.32)] and negatively associated with coronal imaging of tongue muscle stiffness [odds ratio 0.72 (95% confidence interval 0.54–0.95)]. There were no significant differences in tongue stiffness among OSA patients with varying disease severity. SWUE provided a reliable evaluation of tongue muscle stiffness, which appeared to be softer in patients with OSA. Future longitudinal studies are necessary to investigate the relationship between tongue softening and OSA, as well as response to treatment.

## Introduction

Obstructive sleep apnea (OSA) is a breathing disorder characterized by frequent episodes of complete or partial upper airway collapse during sleep (Park et al., 2011). Its prevalence ranges from 9 to 38% in the general population, with increased risks in males, the elderly, and obese individuals (Senaratna et al., 2017). The causes of OSA include abnormal mechanical loading of the pharyngeal wall and disturbed neuromuscular control of the upper airway muscles (Younes, 2003; Patil et al., 2007). An enlarged tongue and/or soft palate may be observed in patients with OSA, which increases breathing resistance and interferes with upper airway patency (Schwab et al., 2003). OSA can lead to intermittent hypoxemia and sleep fragmentation, causing daytime somnolence and also increases the risk for cardiovascular disease (Bradley and Floras, 2009).

The diagnosis of OSA is based on polysomnography (PSG), also called as a sleep study (Young et al., 1997; Kushida et al., 2005). Prompt diagnosis and grading of OSA are essential to design appropriate treatment strategies and the golden standard treatment is continuous positive airway pressure (Cistulli and Grunstein, 2005). In addition, several imaging modalities, such as computed tomography and magnetic resonance imaging (MRI), have been used to assess the upper airway in the OSA population for detection of structural abnormalities (Ahmed and Schwab, 2006; Whyte and Gibson, 2018).

Diagnostic ultrasound (US) has been widely used to assess musculoskeletal disorders in recent decades. It has several benefits, including portability, cost-effectiveness, and zero radiation exposure, and enables dynamic evaluation (Vilanova et al., 2007). Soft tissues of the upper airway can also be clearly evaluated using submental US imaging, which has been used to measure tongue muscle thickness in patients with OSA (Lahav et al., 2009; Shu et al., 2013). Similarly, shear-wave US elastography (SWUE) is an emerging technology used for noninvasive and quantitative evaluation of soft tissue elasticity (Gennisson et al., 2013; Chiu et al., 2020). SWUE estimates tissue elasticity using the shear wave, which propagates along the plane perpendicular to the radiation pulse exerted from the transducer. SWUE has been applied to the evaluation of several musculoskeletal tissues/injuries (e.g., chronic myofascial pain; Shiina et al., 2015). To date, however, there has been a limited number of studies addressing the use of SWUE in patients with upper airway disorders. Accordingly, this study aimed to establish a reliable US protocol for measuring tongue muscle elasticity, as well as to investigate the utility of SWUE in differentiating patients with OSA and those without OSA.

## Materials And Methods

### Subjects

**Figure 1:**
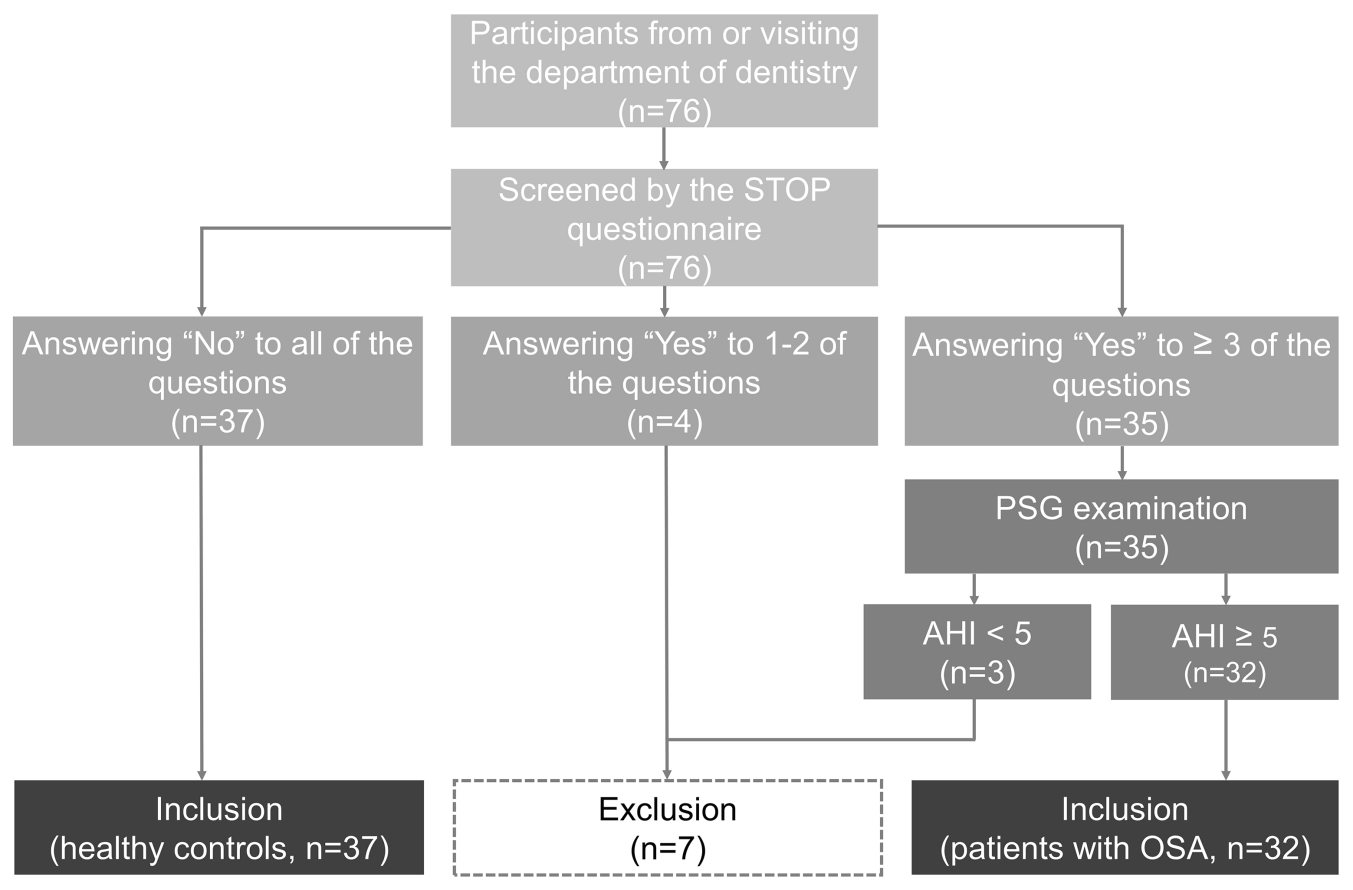
Flow diagram of participant recruitment. OSA, obstructive sleep apnea; PSG, polysomnography; and AHI, Apnea Hypopnea Index.

Between October 2018 and May 2019, 32 OSA patients and 37 healthy subjects were recruited from the department of dentistry. All the subjects were evaluated by the STOP questionnaire, a validated tool to screen the presence of OSA (Chung et al., 2008). The healthy controls were required to answer “No” for all the questions (snoring, daytime tiredness, breathing stop during sleep, and high blood pressure). The participants who answered “Yes” to three or more questions in the STOP questionnaire were further referred for a PSG examination. The OSA patients should have an apnea-hypopnea index (AHI) of ≥5 events/h during sleep, confirmed by a PSG examination within the previous 6 months (Berry et al., 2012a). The flow diagram of participant recruitment is shown in Figure 1. All participants were >20 years of age and their body mass index (BMI) and neck circumference were recorded. The neck circumference was measured in the mid-cervical region with non-stretchable plastic tape while the subjects stood upright by the primary investigator before the PSG examination. Individuals who were pregnant, and those with active infection, the history of radiotherapy or surgery for the head/neck, chronic pulmonary disease or deformity of the oral cavity, and upper airway, were excluded. Potential subjects who took psychiatric medications were also excluded. Informed written consent was obtained from all participants before entry into the study. The research project was approved by the Institutional Review Board of National Taiwan University hospital (IRB number: 20180904RIND).

### PSG Evaluation

Each subject underwent full-night PSG (Embla N7000, Medcare Flaga, Reykjavik, Iceland) in the sleep laboratory in accordance with the OSA diagnosis and management guidelines (Epstein et al., 2009). Multiple channels were incorporated in PSG including electroencephalography, electrooculogram, chin and tibia electromyogram, airflow sensors, electrocardiography, and oxygen meters. PSG was scored by independent sleep technologists and physicians, who were blinded to the US results, in accordance with the 2012 AASM (American Academy of Sleep Medicine) Manual for the Scoring of Sleep and Associated Events (Berry et al., 2012a). AHI, which is derived from the total number of apnea (pause in breathing) and hypopnea (period of shallow breathing, 3% desaturation or an arousal; Berry et al., 2012b) events divided by the total sleep time, was used to classify the severity of sleep apnea as follows: mild (AHI ≥ 5, but <15 events/h), moderate (AHI ≥ 15, but <30 events/h), and severe (AHI ≥ 30 events/h; Duce et al., 2015).

### Ultrasonographic Assessment

#### Device Settings

Submental US images of the tongue muscles were obtained using an US system (Aplio 300 Platinum platform, Toshiba, Tokyo, Japan) using a multi-frequency convex transducer (PVT-375SC, 50 mm wide, 1.5–6 MHz). The scanning depth was set at 8 cm, and the focus at 4 cm. When using the SWUE mode, a color map representing the distribution of tissue elasticity was superimposed on a B-mode image. The assessed rectangular area was required to be homogenously filled with colors ranging from blue (soft) to red (stiff). The propagation mode was displayed simultaneously to demonstrate the contour lines of the radial pulse for ensuring data reliability (Yang et al., 2017). The muscle stiffness was quantified by shear modulus values in kilopascal (kPa). The shear modulus is defined as the ratio of shear stress to the shear strain (Lima et al., 2018). The maximum, minimum, mean, and standard deviation (SD) of shear modulus were calculated for a circular region of interest [ROI (1.5 mm in diameter)] in kPa. The mean values were used for statistical analysis.

### Subject Positioning

Subjects were positioned supine, with slight flexion in the neck and extending the head with a soft pad under the neck to expose the submental region. They were asked to close their mouth with the lips slightly in contact and to hold the breath and swallowing for 10 s during the examination.

### Measurement of Tongue Thickness and Elasticity

**Figure 2:**
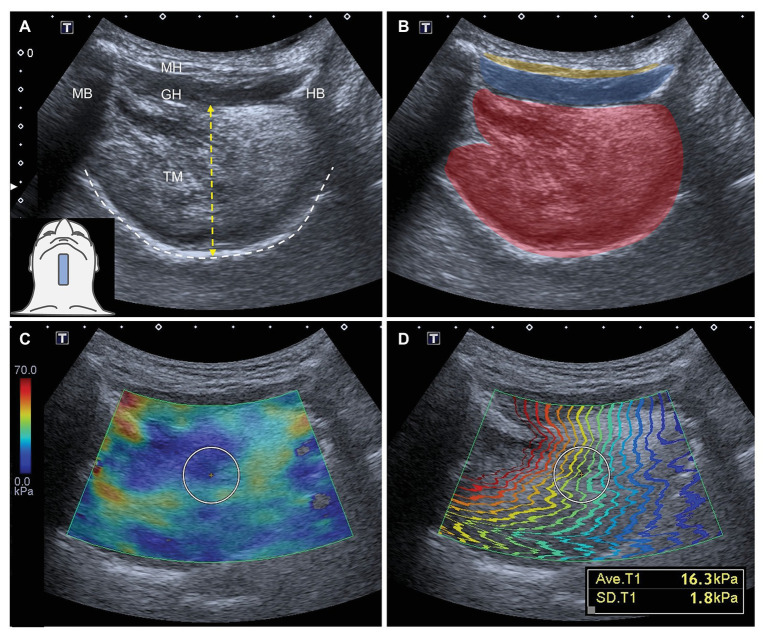
Ultrasound imaging **(A)** and superimposed schematic drawing **(B)** of the tongue muscles in the sagittal plane. The white-dashed line indicates the dorsal surface of the tongue. The length of the yellow-dashed line indicates the thickness of the tongue. The color diagram **(C)** and propagation modes **(D)** of shear wave ultrasound elastography imaging for tongue muscles in the sagittal plane. The average shear modulus of the tongue in the selected region of interest is 16.3 kPa with a standard deviation of 1.8 kPa. TM: tongue muscles, red color block; MH: mylohyoid muscle, yellow color block; GH: geniohyoid muscle, blue color block; MB: mandible; and HB: hyoid bone.

**Figure 3:**
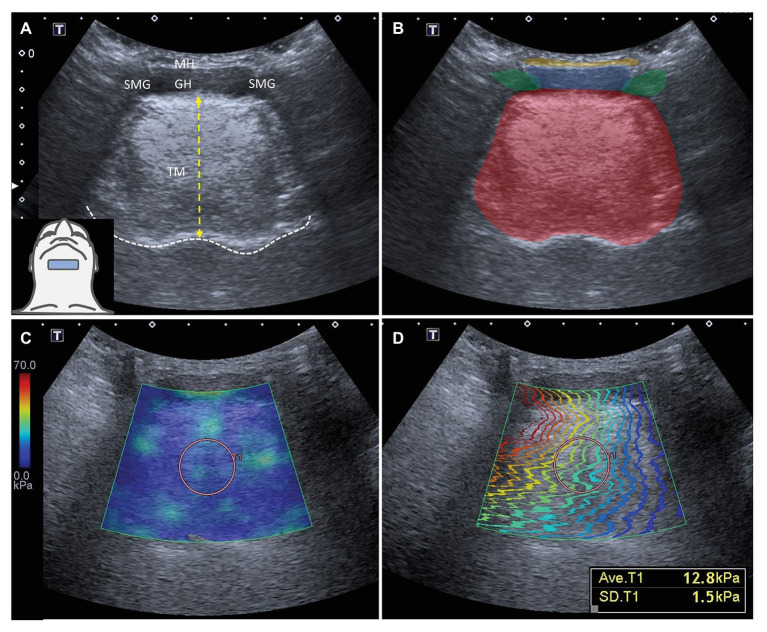
Ultrasound imaging **(A)** and superimposed schematic drawing **(B)** of the tongue muscles in the coronal plane. The white dashed line indicates the dorsal surface of the tongue. The length of the yellow-dashed line indicates the thickness of the tongue. The color diagram **(C)** and propagation modes **(D)** of shear wave ultrasound elastography imaging for the tongue muscles in the coronal plane. The average shear modulus of the tongue in the selected region of interest is 12.8 kPa with a standard deviation of 1.5 kPa. TM: tongue muscles, red color block; MH: mylohyoid muscle, yellow color block; GH: geniohyoid muscle, blue color block; and SMG: submandibular gland, green color block.

All measurements were performed by a single experienced researcher (except for reliability testing), who was not aware of the participants’ diagnosis. The B mode and SWUE images were obtained in the sagittal and coronal views. The transducer was held stationary to lightly contact the skin coupled by a thin layer of gel. To scan the tongue in the sagittal plane, the transducer was placed between the hyoid bone and symphysis of the mandible. The transducer was adjusted to permit the tongue base to appear in the middle of the screen and the acoustic shadow of the symphysis and hyoid bone at both sides of the scanning window. The dorsal surface of the tongue and soft palate were clearly identified (Figures 2A,B). Thereafter, the transducer was rotated to align with the coronal plane and was placed parallel to the anterior portion of hyoid bone with the footprint pointing to the eye corner of the examinee (Figures 3A,B).

Tongue thickness was measured from the deep fascia of the geniohyoid muscle to the thickest part of the dorsal tongue in both the sagittal and coronal planes. Measurements were performed five times using B-mode images and Image J software (National Institutes of Health, Rockville Pike, Bethesda, MD, United States). Tongue stiffness was measured five times in a circular ROI positioned in the center of the tongue, where the contour lines of the shear waves in the propagation mode were the most parallel with the best reliability (Zhou et al., 2017). Their average values were calculated and used in analyses (Figures 2C,D, 3C,D).

### Reliability of Elasticity Measurements

Tongue muscle thickness and elasticity were measured twice in eight healthy volunteers by the primary investigator, with an interval of 24 h between the two examinations. The data were used for calculation of intra-rater reliability. The eight healthy volunteers were also evaluated by the second investigator, and the data were used for computing inter-rater reliability. The intraclass correlation coefficient (ICC) and corresponding 95% confidence interval (CI) were used to quantify the strength of reliability.

### Statistical Analysis

The sample size was calculated based on a study in which magnetic resonance elastography was used to measure tongue elasticity (Brown et al., 2015). The mean (± SD) tongue stiffness was assumed to be 3 ± 0.5 kPa in healthy subjects. Patients with OSA may exhibit a mean decrease of 0.4 kPa in tongue elasticity. The sample ratio was set at 1:1 with a power of 80% and an alpha value of 0.05, thus necessitating the inclusion of 50 subjects in the study.

The Shapiro-Wilk test was used to test the distribution of the obtained data. Normally distributed continuous variables (expressed as mean ± SD) were compared using the independent t-test or the Mann Whitney U test for data that were non-normally distributed. Categorical variables (expressed as number and percentage) were compared using the chi-squared test or Fisher’s exact test in cases of sparse data. Associations between US measurements and the presence of OSA were analyzed using logistic regression and quantified as odds ratio (OR) and corresponding 95% CI. The confounders adjusted in multivariate analysis were selected according to previous studies (Hui et al., 2002; Kim et al., 2004; Sharma et al., 2006; Mirrakhimov et al., 2013) and included age, sex, neck circumference, and BMI. Thereafter, receiver operating characteristic (ROC) curves were plotted to evaluate the diagnostic performance of US parameters for discriminating patients with OSA from those without OSA. The optimal cut-off point for each US parameter was determined according to the Youden index. Additionally, the participants with OSA were divided into three subgroups according to the severity of OSA (Duce et al., 2015). One-way analysis of variance (in case of normal distribution) or the Kruskal-Wallis rank sum test (in case of lacking normal distribution) was used to determine whether there were any between-group differences regarding US measurements. All statistical analyses were performed using Medcalc version 18.2.1 (Broekstraat, Mariakerke, Belgium). The values of *p* were two-sided and differences with *p* < 0.05 were considered to be statistically significant.

## Results

### Intra‐ and Inter-Rater Reliability

The intra-rater reliability values, reflected by ICC, were 0.98 (95% CI, 0.88–1.00) for thickness in the sagittal plane, 0.97 (95% CI 0.84–0.99) for thickness in the coronal plane, 0.90 (95% CI 0.52–0.98) for stiffness in the sagittal plane, and 0.85 (95% CI 0.27–0.97) for stiffness in the coronal plane. The ICC values of inter-rater reliability were 0.95 (95% CI 0.74–0.99) for thickness in the sagittal plane, 0.92 (95% CI 0.62–0.98) for thickness in the coronal plane, 0.84 (95% CI 0.20–0.97) for stiffness in the sagittal plane, and 0.84 (95% CI 0.20–0.97) for stiffness in the coronal plane. Overall, the reliability measurements were classified as “excellent” for tongue muscle thickness and “good” for tongue stiffness (Shrout and Fleiss, 1979).

### Participant Characteristics and US Measurements

Comparative data are summarized in Table 1. There was no significant difference in age between the two groups, although the OSA group had more males (*p* = 0.022) and exhibited higher BMI values (*p* = 0.019). The data of PSG in the OSA group are detailed in Table 2.

**Table 1:** Basic characteristics and ultrasound measurement of tongue in participants with or without obstructive sleep apnea (OSA).

	OSA (*n* = 32)	Non-OSA (*n* = 37)	*p*
Baseline characteristics			
Age	53.06 ± 12.77 (48.46–57.67)	47.59 ± 15.50 (42.43–52.76)	0.118
Male gender (%)	25(78.13%)	19 (51.35%)	0.022^*^
Neck circumference (cm)	37.45 ± 3.11 (36.31–38.60)	35.30 ± 3.73 (34.04–36.56)	0.014^*^
Body mass index (kg/m^2^)	25.62 ± 3.84 (24.24–27.01)	23.46 ± 3.55 (22.26–24.67)	0.019^*^
**Ultrasound measurements**			
Tongue thickness (mm, sagittal)	46.88 ± 5.26 (44.98–48.77)	42.49 ± 6.00 (40.49–44.49)	0.002^*^
Tongue thickness (mm, coronal)	48.40 ± 5.55 (46.40–50.40)	42.42 ± 5.84 (40.47–44.37)	<0.001^*^
Tongue stiffness (kPa, sagittal)	19.90 ± 5.79 (17.81–21.99)	20.52 ± 6.68 (18.29–22.74)	0.613
Tongue stiffness (kPa, coronal)	11.34 ± 1.97 (10.63–12.05)	14.05 ± 4.01 (12.71–15.38)	0.006^*^

All continuous data are expressed as mean ± standard deviation (95% confidence interval). All categorical data are expressed as number (percentage).

* Indicates *p* < 0.05.

**Table 2:** Data of polysomnography in participants with obstructive sleep apnea.

Parameter	Mild OSA (*n* = 4)	Moderate OSA (*n* = 18)	Severe OSA (*n* = 10)	*p* value
Age (years)	53.25 ± 17.54	52.11 ± 12.15	54.70 ± 13.24	0.883
Male participants (%)	4(100%)	12(67%)	9(90%)	0.203
Epworth sleepiness scale score	14.33 ± 8.62	9.39 ± 3.86	10.22 ± 3.27	0.195
Body mass index (kg/m^2^)	25.05 ± 2.66	26.25 ± 4.54	24.72 ± 2.75	0.586
Neck circumference (cm)	38.75 ± 2.63	37.39 ± 3.76	37.00 ± 1.66	0.656
**Polysomnographic results**				
Total sleep time (min)	274.80 ± 66.34	327.11 ± 56.63	328.69 ± 28.67	0.250
Sleep efficiency (%)	75.23 ± 13.61	86.40 ± 12.72	87.13 ± 6.76	0.268
AHI (events/h)	10.48 ± 2.76	21.64 ± 4.32	43.88 ± 10.84	<0.0001^*^
OAI (events/h)	2.43 ± 2.14	9.02 ± 5.63	27.51 ± 11.75	<0.0001^*^
RDI (events/h)	10.62 ± 3.01	23.26 ± 4.66	44.26 ± 10.61	<0.0001^*^
AHI REM (events/h)	30.97 ± 14.28	35.98 ± 17.55	41.33 ± 15.60	0.596
% REM	11.60 ± 3.32	20.09 ± 7.02	17.68 ± 7.06	0.144
Arousal index (events/h)	10.43 ± 0.76	13.36 ± 6.13	18.62 ± 10.06	0.148
SpO2 awake (%)	95.93 ± 1.62	96.04 ± 1.45	95.80 ± 1.44	0.923
Desaturation index (events/h)	10.17 ± 3.89	19.84 ± 5.46	38.91 ± 8.14	<0.0001^*^
Lowest oxygen saturation (%)	85.33 ± 4.16	80.50 ± 5.61	77.11 ± 11.35	0.266
Saturation <90% (%)	1.43 ± 1.29	1.92 ± 3.24	7.13 ± 8.24	0.053

OSA, obstructive sleep apnea; AHI, apnea-hypopnea index; OAI, obstructive apnea index; RDI, respiratory disturbance index; AHI REM, apnea-hypopnea index in rapid eye movement (REM) sleep stage; % REM, percentage of REM sleep stage, SaO2 awake, average oxygen saturation during wakefulness. All data are expressed as the mean ± standard deviation or number (percentage).

* Indicates *p* < 0.05.

**Figure 4:**
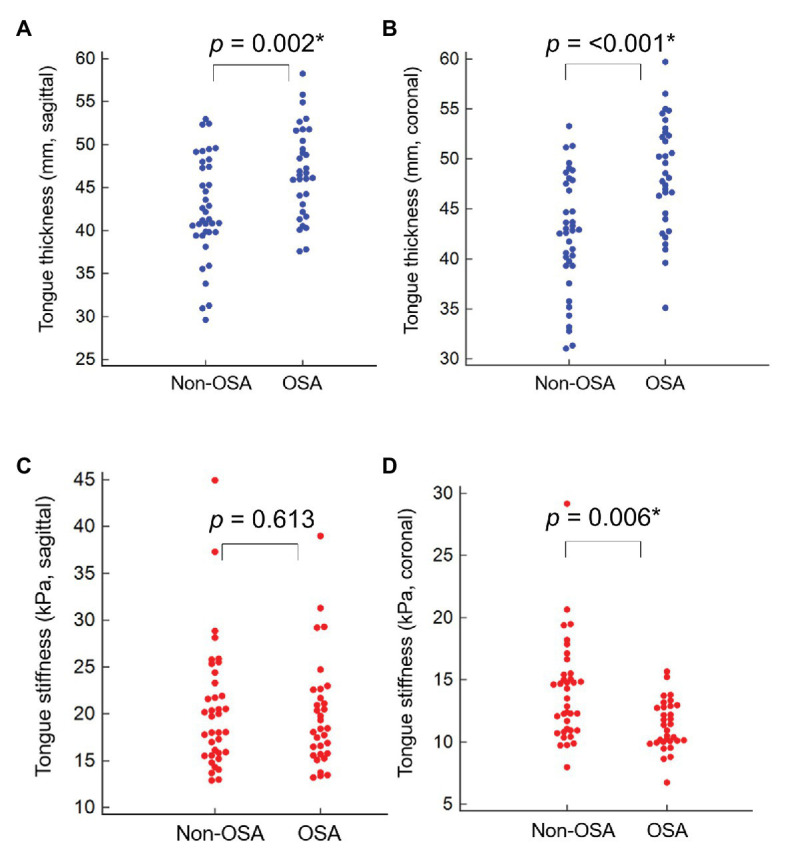
The dot plots demonstrated the individual measurements of tongue muscle thickness in the sagittal **(A)** and coronal **(B)** views and tongue muscle stiffness in the sagittal **(C)** and coronal **(D)** views. OSA, obstructive sleep apnea.

Regarding US measurements, OSA patients exhibited significantly thicker tongues in both the sagittal and coronal views, with lower shear modulus (kPa) in the coronal plane. The distributions of all the individual US measurements were demonstrated on the dot plots (Figure 4). Associations between tongue muscle thickness/stiffness and OSA are presented in Table 3. In univariate analysis, OSA was associated with increased tongue thickness in the sagittal [OR 1.15 (95% CI 1.04–1.27)] and coronal [OR 1.21 (95% CI 1.09–1.34)] views. Furthermore, OSA had an inverse association with the shear modulus (kPa) of tongue muscles [OR 0.70 (95% CI 0.56–0.88)] in the coronal view. Regarding multivariate analysis adjusted for age, sex, neck circumference, and BMI, an increase in tongue muscle thickness [OR 1.16 (95% CI 1.01–1.32)] and a decrease in shear modulus of tongue muscles in the coronal view [OR 0.72 (95% CI 0.54–0.95)] were associated with the presence of OSA. However, tongue stiffness in the sagittal view did not demonstrate any association with the disease.

**Table 3:** Association between obstructive sleep apnea and ultrasound measurements of tongue thickness and stiffness.

	**Univariate analysis**		**Multivariate analysis**	
	Odds ratio (95%CI)	*p*	Odds ratio (95%CI)	*p*
Tongue thickness (mm, sagittal)	1.15 (1.04, 1.27)	0.0045^*^	1.05 (0.92, 1.20)	0.4458
Tongue thickness (mm, coronal)	1.21 (1.09, 1.34)	0.0005^*^	1.16 (1.01, 1.32)	0.0299^*^
Tongue stiffness (kPa, sagittal)	0.98 (0.91, 1.06)	0.6786	0.98 (0.89, 1.07)	0.5902
Tongue stiffness (kPa, coronal)	0.70 (0.56, 0.88)	0.0023^*^	0.72 (0.54, 0.95)	0.0214^*^

In the multivariate analysis, the model was adjusted by age, gender, neck circumference, and body mass index.

* Indicates *p* < 0.05.

**Figure 5:**
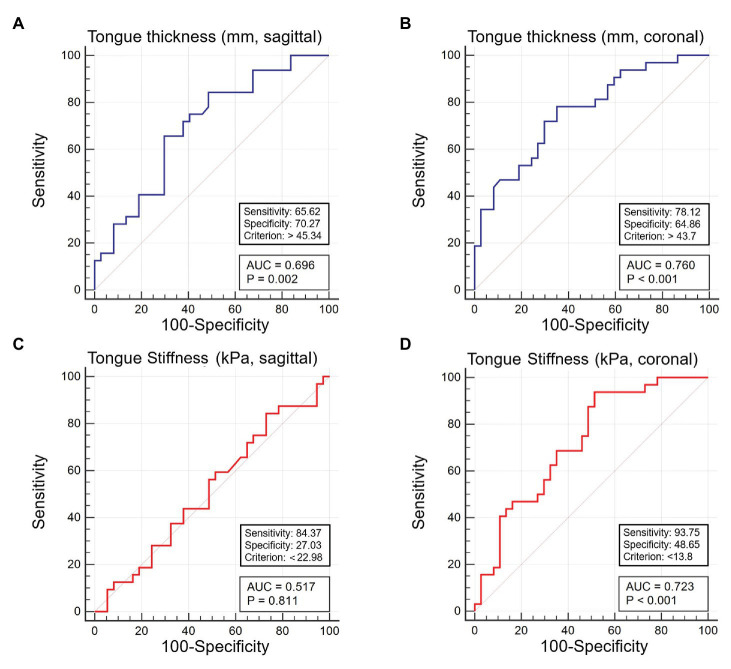
Receiver operating characteristic curve analysis for ultrasound measurements of tongue thickness in the sagittal **(A)** and coronal **(B)** views, and tongue elasticity in the sagittal **(C)** and coronal **(D)** views for discriminating participants with and without obstructive sleep apnea.

The distinctive capabilities of US thickness and stiffness measurements for OSA are presented in Table 4 and Figure 5. The areas under the ROC curve for tongue thickness in the sagittal view, tongue thickness in the coronal view, tongue stiffness in the sagittal view, and tongue stiffness in the coronal view were 0.696 (95% CI 0.572–0.819), 0.760 (95% CI 0.642–0.854), 0.517 (95% CI 0.393–0.639), and 0.723 (95% CI 0.602–0.824), respectively. The optimal cut-off values for tongue thickness in the sagittal and coronal views, and tongue stiffness in the sagittal and coronal views for distinguishing OSA were 45.34 mm (sensitivity 65.6% and specificity 70.3%), 43.70 mm (sensitivity 78.1% and specificity 64.9%), 22.98 kPa (sensitivity 84.4% and specificity 27.0%), and 13.80 kPa (sensitivity 93.8% and specificity 48.7%).

**Table 4:** Receiver operating characteristic curve analysis for the best cut-off points as regards ultrasound measurements for differentiating patients with obstructive sleep apnea.

	Threshold	Sensitivity (95% CI)	Specificity (95% CI)	Area under curve (95%CI)	*p*
Tongue thickness (mm, sagittal)	>45.34	65.62% (46.8–81.4%)	70.27% (53.0–84.1%)	0.696 (0.572–0.819)	0.002^*^
Tongue thickness (mm, coronal)	>43.70	78.12% (60.0–90.7%)	64.86% (47.5–79.8%)	0.760 (0.642–0.854)	<0.001^*^
Tongue stiffness (kPa, sagittal)	<22.98	84.37% (67.2–94.7%)	27.03% (13.8–44.1%)	0.517 (0.393–0.639)	0.811
Tongue stiffness (kPa, coronal)	<13.80	93.75% (79.2–99.2%)	48.65% (31.9–65.6%)	0.723 (0.602–0.824)	<0.001^*^

CI, confidence interval.

* Indicates *p* < 0.05.

Subgroup analysis was performed to examine the association between OSA severity and tongue muscle thickness/elasticity (Table 5) in the OSA group. There were no differences among the subgroups in this regard.

**Table 5:** Associations between disease severity and ultrasound measurements in patients with obstructive sleep apnea (OSA).

	Mild OSA (*n* = 4)	Moderate OSA (*n* = 18)	Severe OSA (*n* = 10)	*p*
Tongue thickness (mm, sagittal)	44.05 ± 5.46	47.97 ± 5.39	46.05 ± 4.87	0.347
Tongue thickness (mm, coronal)	46.52 ± 6.03	49.20 ± 5.83	47.73 ± 5.13	0.630
Tongue stiffness (kPa, sagittal)	18.33 ± 4.20	20.06 ± 6.63	20.23 ± 5.03	0.852
Tongue stiffness (kPa, coronal)	11.71 ± 1.66	11.61 ± 1.80	10.71 ± 2.33	0.493

## Discussion

This study aimed to assess tongue muscle stiffness in OSA patients using SWUE. First, we demonstrated that the stiffness of tongue muscles could be reliably measured using SWUE. Second, the US measurements demonstrated that OSA patients tended to have thicker and softer tongue muscles. Third, the thickness and stiffness of tongue muscles did not vary according to OSA severity.

Our findings revealed that the reliability of US measurement ranged from 0.92 to 0.98 for tongue thickness and from 0.84 to 0.90 for tongue stiffness. Although several studies have investigated tongue thickness using US (Lahav et al., 2009; Shu et al., 2013; Liao et al., 2016), only one examined its reliability. In eight healthy volunteers, Shu et al. (2013) reported good intra‐ and inter-observer ICCs for variation (range, 2.3–3.0%) in tongue thickness measurements. In our study, we standardized the position of patients and the ROI to minimize variation in muscle thickness and stiffness during the examination (Thoirs and English, 2009).

To the best of our knowledge, this study is the first to validate the reliability of US measurements for tongue muscle stiffness. Unlike strain elastography (Hsu et al., 2020), which quantifies structure displacement under the stress exerted by the operator, SWUE estimates tissue elasticity through measuring velocity of shear waves propagating laterally from the target after receiving acoustic radiation generated by the transducer (Gennisson et al., 2013). Therefore, in our study, the measurement of tongue muscle stiffness also benefited from less vulnerability to variation with regard to manual compression force applied during elastography.

As SWUE was used in the present study, there was no need for the investigator to perform manual compression during the elasticity measurement. Therefore, this advantage allows the investigator to place the transducer and position the patient exactly like the protocol of most sub-mental US measurements (Shu et al., 2013). The only point requiring extra attention is that the transducer should be kept with light touch on the skin to prevent the underlying tissue from deformation while using SWUE. In other words, if the investigators are aware of the protocol of routine submental ultrasonography, they can conduct SWUE measurements of tongue muscles confidently after familiarization with the machine setting. Of course, a short period of training under the supervision of the experienced operator is highly suggested to ensure the reliability of measurements.

Our study yielded increased tongue muscle thicknesses in patients with OSA compared with healthy subjects. Enlargement of tongue volume narrows the oral cavity and pharyngeal space and has been shown to be associated with OSA (Schwab et al., 2003; Schwartz et al., 2008). Schwab et al. (2003) used MRI to evaluate the upper airway of patients with OSA. The authors demonstrated that patients with OSA had larger tongue volumes and thicker pharyngeal walls. The above-mentioned association also remained after adjustment for age, sex, race, craniofacial size, and parapharyngeal fat. Liao et al. (2016) studied 66 Asian OSA patients using US imaging and, consistent with our findings, reported that tongue base thickness was the only independent predictor of severe OSA. Herewith, the cut-off values for tongue thickness (60.0 mm) for defining the risk for OSA in their study was different/higher from those of ours in the sagittal (45.3 mm) and coronal (43.7 mm) views (Liao et al., 2016). Of note, in that study, the subcutaneous tissues, and mylohyoid and geniohyoid muscles were also included in the measurements. Our research also revealed that subjects with and without OSA could possibly be differentiated according to tongue muscle thickness, with sensitivity ranging from 65.62 to 78.12% and specificity ranging from 64.86 to 70.27%.

According to our results, the tongue tissue of OSA patients appeared to be softer in the coronal view. This finding is consistent with a previous study, in which magnetic resonance elastography revealed that the shear modulus (kPa) of tongue tissue was approximately 10% lower in nine OSA patients compared with healthy controls (Brown et al., 2015). Furthermore, Kim et al. (2014) performed a prospective cross-sectional study using MRI to measure the tongue volume. The authors also found an increase in fatty infiltration at the tongue base in OSA patients. To this end, OSA may develop due to the proliferation of adipose tissue around the airway, which softens the tongue muscle. Because our SWUE findings were also similar, tongue stiffness measured under the coronal view – in addition to tongue muscle thickness – can potentially be used to differentiate subjects with OSA from those without OSA.

In our study, the significant association between tongue stiffness and OSA was only found when SWUE was performed in the coronal – but not sagittal – view. Cortez et al. (2016) evaluated the reliability of SWUE measurements on normal skeletal muscles and reported better reproducibility in the longitudinal than in the transverse plane. However, they assessed the gastrocnemius and anterior tibialis muscles, where the pennate structure is unidirectional and parallel (Cortez et al., 2016). In contrast, the human tongue is composed of four extrinsic and four intrinsic muscles that have different fiber alignments relative to the tongue surface (Takemoto, 2008). In this sense, the discrepancy of fiber trajectory was easier to present in the sagittal than in the coronal view, thus leading to more anisotropy (of the target) during imaging. Therefore, the increase in anisotropy may further contribute to variations among measurements, which may possibly explain the similar sagittal plane elasticity values in subjects with and without OSA.

Results of our study also revealed that tongue muscle stiffness did not vary according to the severity of OSA, which limited the usefulness of SWUE in aiding differential diagnosis. Patil et al. (2007) measured upper airway collapsibility in patients with OSA and reported that disease severity was not only related to the mechanical load of the upper airway but also to blunted neuromuscular response. Brown et al. (2013) studied respiratory movements of the upper airway using dynamic MRI and found blunted tongue motions in patients with OSA. The two aforementioned studies demonstrate that the decreased muscle tension of the tongue is not the only factor in OSA (Patil et al., 2007; Brown et al., 2013). Nevertheless, reduced central respiratory drive and the malposition of the structures in the upper airway both contribute to the development of OSA. Another possibility is that the tongues of OSA patients would likely soften over time without symptom deterioration. However, because our study had a cross-sectional design, it was not possible to examine this hypothesis. There is also necessity for a long-term study to investigate how aging may affect tongue mechanical properties. Furthermore, the factors associated with OSA are miscellaneous, comprising the sensitivity of ventilatory control (i.e., loop gain), respiratory arousal threshold, pharyngeal collapsibility, and compensatory muscle responsiveness (Sands et al., 2018a,b). All the aforementioned mechanisms could lead to variability of US measurements, resulting in no correlation of tongue muscle stiffness with OSA severity.

A recently published similar study (Chang et al., 2020) reported results opposite to ours, revealing the tongue muscles having higher stiffness in patients with OSA than healthy controls. The architecture of the tongue is complex due to interdigitating intrinsic and extrinsic muscles. Recently, the anatomical details of tongue muscles have been appreciated by the development of the super-resolution volume reconstruction method (Woo et al., 2012), establishment of a spatial-temporal atlas during speech and swallowing (Woo et al., 2015) and the segmented analysis of different muscle fibers on MRI imaging (Stone et al., 2018). Furthermore, the increase of fat depositing in the tongue base of obese and aging patients (Kim et al., 2014), regional differences in proportion and diameter of muscle fiber types (Stål et al., 2003) as well as the size and collagen composition of the lingual frenulum (Guilleminault et al., 2016; Mills et al., 2020) would be related to the stiffness measurement in patients with OSA. In addition, we found that the age distribution of the participants in their study (Chang et al., 2020) was different from that of ours. In their study, the average age was significantly lower in the control group than in the patient group. In our study, no significant between-group age difference existed. The points mentioned above could be the potential cause of the difference between the two study results.

**Figure 6:**
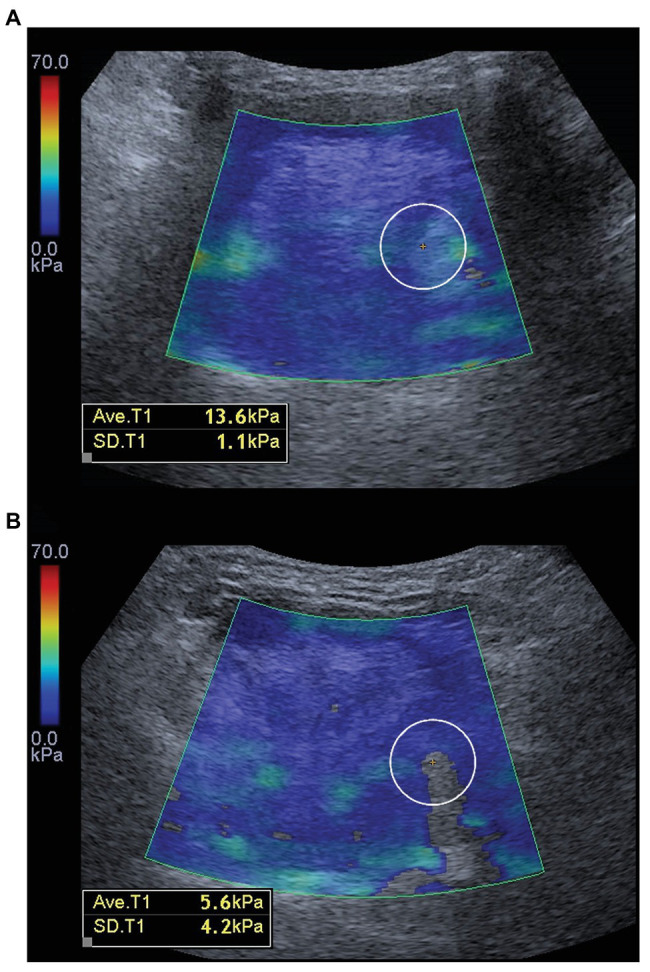
A color filling defect in the elastogram **(A)** can lead to underestimation of the actual stiffness **(B)** of the target tissue.

In the study by Chang et al. (2020), reliability testing was not done before enrollment of the participants. In their published data, some of the elastogram boxes appeared to have color filling defects. This point might technically influence the representativeness of elasticity. Taking Figure 6 as an example, if there is no filling defect inside the color box, the kPa value is 13.6. However, if a small filling defect exists inside the color box, the kPa value drops to 5.6. The difference could be up to 2.61 times in the same muscle. In our study, we had confirmed that all the elastogram boxes were filled with color during the examination, and our result was consistent with the antecedent MRI study (Brown et al., 2015), demonstrating softer tongue muscles in patients with OSA than healthy controls. Another strength of our study was that the association between the US measurements and the presence of OSA had been adjusted by age, sex, neck circumference, and BMI using the multivariate logistic regression.

Our study has several clinical implications. First, considering the conflicting results of tongue muscle stiffness from two available papers (Brown et al., 2015; Chang et al., 2020), ours arises to be an important reference to determine the true relationship between OSA and tongue stiffness. Second, our study revealed good reliability of muscle stiffness measurements using SWUE. However, our study found that the tongue stiffness seemed to have higher sensitivity but lower specificity than tongue thickness for the diagnosis of OSA in the coronal view (Table 4). Therefore, SWUE (possibly with better sensitivity) and B-mode submental ultrasonography (possibly with better specificity) have their own pros and cons in the evaluation of OSA. Furthermore, tongue thickness/stiffness cannot be used alone as a diagnostic tool for OSA concerning a considerable overlap of US measurements between the OSA and non-OSA groups in our study. In the future, owing to the accessibility and portability of US machines, it might be possible to combine the findings from questionnaires, B-mode imaging and SWUE to generate a composite score as a screening tool for OSA. Third, as the tongue muscle stiffness was confirmed to reduce in patients with OSA, we would like to know whether oropharyngeal exercises can ameliorate the symptoms of OSA. Hence, clinical trials focusing on tongue exercises can also be developed to see whether the US elasticity measurements improve after intervention.

There were several limitations to this study that should be acknowledged. First, all subjects were examined while they were awake. As such, whether tongue stiffness changed during sleep could have not been determined in our protocol. Second, the healthy controls did not receive PSG examinations. However, they were required to answer “No” in all the questions in the STOP questionnaire. As answering “Yes” to 2 or fewer than 2 questions is considered to be low risk for OSA, the prevalence of asymptomatic OSA in our control group should be trivial. Furthermore, a separate additional analysis – to compare the US measurements between control subjects with and those without PSG findings – revealed no significant between-group difference (Supplementary Table 1). Third, our study used a cross-sectional design and the case number is relatively small; therefore, the causal relationship between decreased tongue elasticity and OSA needs to be validated in large longitudinal cohort trials.

## Conclusion

Our findings suggested that the tongues of OSA patients were thicker and softer and the stiffness of tongue muscles did not vary according to the severity of OSA. Therefore, future longitudinal studies are necessary to explore causation between tongue thickening/softening and OSA; nevertheless, SWUE appears to be a reliable tool for evaluating the stiffness of tongue muscles.

## Data Availability Statement

The original contributions presented in the study are included in the article/Supplementary Material, further inquiries can be directed to the corresponding author.

## Ethics Statement

The studies involving human participants were reviewed and approved by the Institutional Review Board of National Taiwan University Hospital (IRB number: 20180904RIND). The patients/participants provided their written informed consent to participate in this study.

## Author Contributions

K-VC, C-AC, and Y-JC conceived and designed the study, recruited the study subjects, and planned and performed the statistical analysis. W-TW, C-AC, Y-JC, K-VC, and LÖ contributed to study supervision and critical revision of the manuscript. All authors have read and approved the final manuscript.

### Conflict of Interest

The authors declare that the research was conducted in the absence of any commercial or financial relationships that could be construed as a potential conflict of interest.
