# Supplementary material for: Reliability of Sonoelastography Measurement of Tongue Muscles and Its Application on Obstructive Sleep Apnea
Source: Front Physiol. 2021 Mar 25;12:654667. doi: 10.3389/fphys.2021.654667 (PMC8027470; doi:10.3389/fphys.2021.654667)
Supplement: Supplementary file 1 [file Table_1.docx]

**Supplementary Table 1.** Comparison between control subjects with and those without polysomnography (PSG) findings.

| **Parameter** | **Without PSG**  **(n=34)** | **With PSG**  **(n=3)** | ***p* value** |
| --- | --- | --- | --- |
| **Ultrasound measurement** | | | |
| Tongue thickness  (mm, sagittal) | 42.53 ± 6.23 | 41.71 ± 3.25 | 0.824 |
| Tongue thickness  (mm, coronal) | 42.62 ± 6.03 | 42.71 ± 2.18 | 0.979 |
| Tongue stiffness  (kPa, sagittal) | 20.73 ± 6.91 | 21.78 ± 5.39 | 0.800 |
| Tongue stiffness  (kPa, coronal) | 14.06 ± 4.13 | 12.37 ± 1.96 | 0.490 |
| **Polysomnography data** | | | |
| Total sleep time (min) | N.A. | 397.33 ± 51.75 | N.A. |
| Sleep efficiency (%) | N.A | 93.90 ± 0.96 | N.A. |
| AHI (events/h) | N.A. | 1.60 ± 0.61 | N.A. |
| OAI (events/h) | N.A. | 0.10 ± 0.17 | N.A. |
| RDI (events/h) | N.A. | 1.86 ± 0.42 | N.A. |
| AHI REM (events/h) | N.A. | 2.73 ± 0.90 | N.A. |
| % REM | N.A. | 24.20 ± 4.65 | N.A. |
| Arousal index (events/h) | N.A. | 8.73 ± 1.50 | N.A. |
| SpO2 awake (%) | N.A. | 96.97±1.05 | N.A. |
| Desaturation index (events/h) | N.A. | 1.87 ± 2.08 | N.A. |
| Lowest oxygen saturation (%) | N.A. | 92.00±3.00 | N.A. |
| Saturation < 90% (%) | N.A. | 0.00 ± 0.00 | N.A. |

OSA = obstructive sleep apnea, AHI = apnea-hypopnea index, OAI = obstructive apnea index, RDI=respiratory disturbance index; AHI REM = apnea-hypopnea index in the rapid eye movement (REM) sleep stage, % REM = percentage of REM sleep stage, SaO2 awake = average oxygen saturation during wakefulness. N.A.: not applicable. All data are expressed as the mean ± standard deviation or number (percentage). The PSG data of the three control subjects were retrieved from their recent annual health examination.

* Indicates p<0.05
